# Editorial: Advances in nanotechnology for the removal and detection of emerging contaminants from water

**DOI:** 10.3389/fchem.2025.1540487

**Published:** 2025-01-31

**Authors:** Gayani Pathiraja

**Affiliations:** Department of Nanoscience, Joint School of Nanoscience and Nanoengineering, University of North Carolina at Greensboro, Greensboro, NC, United States

**Keywords:** nanotechnology, emerging contaminants, removal, detection, nanomaterials, water treatment methods

Nanotechnology is a potential strategy for addressing the challenges of emerging contaminants (ECs) in water sources. These contaminants, including pharmaceuticals, pesticides, personal care products, heavy metals, poly-fluoroalkyl substances, plastics, and biological pollutants, often raise the requirements of innovative solutions for their detection and removal. Over the past decade, advanced nanomaterials, including metal oxides, metals, quantum dots, metal-organic frameworks nanostructures, carbon nanotubes, graphene, and nanocomposites, have significantly enhanced the sensitivity and specificity of contaminant detection, offering a cost-effective solution for water purification. Although technological and material advancements are progressing, there are challenges associated with the environmental and health impacts of the development of nanomaterials. Therefore, nowadays, current research focuses on developing green methodologies to fabricate nanomaterials with less toxicity. The lower cost and scalability to cover regulatory aspects also became the top key factors in integrating nanotechnology into environmental remediation. Numerous original articles were submitted alongside several reviews on the current Research Topic, showcasing the latest advancements in nanomaterials-based removal and detection methods for emerging contaminants in water.

The review article “*Unveiling the contemporary progress of graphene-based nanomaterials with a particular focus on the removal of contaminants from water: a comprehensive review*” by Assad et al. describes the contribution of two-dimensional graphene nanomaterial derivatives, including graphene oxide (GO), reduced graphene oxide, and functionalized graphene, to removing pollutants in water treatment. They have highlighted the unique properties of graphene structures, such as its high specific surface area and exceptional mechanical, electrical, and chemical characteristics, with examples that make it a promising candidate for removing various pollutants from water sources.

However, the authors address the challenges of using these nanomaterials for practical applications and urge the potential toxicity of graphene and safety assessments to protect both humans and the environment. They advocate for interdisciplinary approaches to optimize graphene derivatives for emerging contaminants’ decontamination.

The study by Faisal et al. introduces an electrochemical sensor designed for efficient nitrite detection in aqueous solutions. This sensor utilizes a glassy carbon electrode (GCE) modified with a nanocomposite comprising gold nanoparticles (Au-NPs), polypyrrole carbon (PPyC), and strontium titanate (SrTiO₃), synthesized via ultrasonication. Their electrochemical sensor has demonstrated a linear response to nitrite concentrations ranging from 0.15 to 1.5 mM, with a sensitivity of 0.5 μA/μM.cm^2^ and the detection limit is 20.00 ± 1.00 µM. The sensor exhibited satisfactory stability and reliability under ambient conditions. The successful detection of nitrates in real samples further confirms the practical applicability of the sensor for monitoring environmental samples. This work presents a novel approach to fabricating a novel composite that can extend the electrochemical detection of other types of halogens in water.

Nanomaterials offer sustainable methods that contribute to the development of environmentally friendly solutions for treating dye-contaminated wastewater. One such example is a green synthesis approach presented by Lal et al. with the production of ZnO nanoparticles using *Bombax ceiba* leaf extract as an eco-friendly reducing and stabilizing agent that shows significant photocatalytic efficiency in degrading toxic dyes. These hexagonal crystalline structures of ZnO nanoparticles with an average size of 17 nm have achieved 99% degradation of methylene blue and 79% of methyl orange under sunlight. This research highlights the potential of these nanoparticles for environmental remediation, particularly in water treatment, showcasing sustainability and cost-effectiveness as pivotal features of this biogenic synthesis method.

Heavy metals are another class of emerging contaminants in aquatic systems. Their persistence, even at trace concentrations, poses significant environmental and human health risks. Alhaithloul et al. explores the development of a novel nanocomposite consisting of guar gum/polyvinyl alcohol/montmorillonite films using a green synthesis method for efficient removal of heavy metal ions, specifically Cu^2^⁺ and Cd^2^⁺, from aqueous solutions. This nanocomposite exhibited excellent thermal stability and high adsorption capacities, achieving equilibrium within 4 h. The adsorption mechanism of this composite follows a pseudo-second-order kinetic model, indicating the chemical adsorption mechanism. The films demonstrated high reusability, with regeneration efficiency exceeding 95% using EDTA. This work highlights the potential of sustainable polymer-based nanocomposites for heavy metals removal and wastewater treatment.

The toxicological profiles of persistent pesticides, including paraquat and others, accumulate in food chains, leading to magnified toxic effects in higher organisms, including humans. The article by Baigorria et al. presents the synthesis and characterization of cyclodextrin-silica (CDSi) hybrid nanocomposites as advanced materials for the removal of paraquat and other pesticides from water. Through an environmentally friendly process, the composites (α-CDSi, β-CDSi, γ-CDSi) were designed to optimize adsorption efficiency. Adsorption studies revealed rapid and effective removal capacities, with α-CDSi achieving the highest efficiency at 87.36 mg/g. The thermodynamic analyses confirmed the rapidness and exothermic nature of the adsorption process, governed primarily by physisorption mechanisms. These findings underscore the potential of CDSi composites as sustainable and effective solutions for addressing pesticide contamination in water resources. Moreover, Adesanmi et al. introduces electrospun carbon nanofibers (ECNFs) as an efficient adsorbent for the removal of organophosphate pesticides from aqueous solutions. The fibers are engineered with a high surface area and functional groups to enhance adsorption efficiency. Adsorption experiments demonstrated rapid uptake and high removal capacities under optimized conditions for the removal of ethion pesticide. Thermodynamic analysis revealed that the adsorption process is exothermic and spontaneous, driven primarily by physisorption with some chemisorption contributions. The ECNFs exhibit high reusability, maintaining performance over multiple cycles, making them a promising and sustainable material for addressing pesticide contamination in water.

